# Meningeal carcinomatosis underdiagnosis and overestimation: incidence in a large consecutive and unselected population of breast cancer patients

**DOI:** 10.1186/s12885-015-2042-y

**Published:** 2015-12-29

**Authors:** Gloria Mittica, Rebecca Senetta, Lorenzo Richiardi, Roberta Rudà, Renato Coda, Isabella Castellano, Anna Sapino, Paola Cassoni

**Affiliations:** 1 Division of Medical Oncology, University of Turin Medical School, Fondazione del Piemonte per l’Oncologia-Institute for Cancer Research and Treatment of Candiolo, Strada Provinciale 142 km 3,95, 10060 Candiolo, Turin Italy; 2 Department of Medical Sciences, University of Turin, Via Santena 7, 10126 Turin, Italy; 3 Department of Neuro-Oncology, University and City of Health and Science Hospital, Via Cherasco 15, 10126 Turin, Italy

**Keywords:** Meningeal carcinomatosis, Breast cancer, Incidence

## Abstract

**Background:**

The incidence of meningeal carcinomatosis appears to be higher than in the past due to advances in neuro-imaging diagnostic techniques and improvements in cancer survival. Among solid tumors, breast cancer is the cancer most commonly associated with meningeal carcinomatosis, with an incidence rate of between 0.8 and 16 %. Aim of this study has been i) to evaluate the incidence of meningeal carcinomatosis in a continuous breast cancer unselected series treated in a dedicated Breast Unit and ii) to define the clinico-pathological and molecular parameters associated with meningeal carcinomatosis development.

**Methods:**

A retrospective series of 1915 consecutive patients surgically treated for breast cancer between 1998 and 2010 was collected. Clinico-pathological data were recorded from medical charts and pathological reports, including the date of development of symptomatic meningeal carcinomatosis. Meningeal carcinomatosis incidence was determined at both 5- and 10-year follow-ups.

**Results:**

Three patients in the first 5 years of follow-up and six patients in 10 years of follow-up developed meningeal carcinomatosis. An incidence rate of 5.44 per 10,000 patients (95 % CI: 1.75–16.9) was observed, with a 5-year risk of 0.3 %. At 10-year follow up, the rate increased to 7.55 per 10,000 patients (95 % CI: 3.39–16.8). In a univariate analysis, young age, tumor size larger than 15 mm, histological grade 3, more than three metastatic lymph nodes, negative estrogen receptor, positive HER2 and high proliferative index were significantly associated with meningeal carcinomatosis development.

**Conclusions:**

In an unselected breast cancer population, meningeal carcinomatosis is a rare event that is associated with adverse prognostic factors. Meningeal carcinomatosis incidence is overestimated when recorded in biased/high-risk selected breast cancer patients and should not be considered to accurately reflect the overall breast cancer population.

## Background

Meningeal carcinomatosis (MC) is caused by the spread of cancer cells to the leptomeninges and by their dissemination within the cerebrospinal fluid (CSF). MC has been reported to occur in 5–10 % of all solid tumors, mainly in breast cancer (BC), lung cancer and malignant melanoma in adult patients [1, 2]. In particular, MC represents a well-known complication in BC [3–5]; in recent years, its occurrence has increased in this patient setting, mainly due to advances in neuro-imaging techniques and to remarkable improvements in BC survival. However, to date, few studies have described the true incidence of MC in BC patients, and the results have proved controversial. In fact, the reported estimates of MC occurrence vary considerably, ranging from 0.8 to 6.6 % in clinical reports to 2.6–16 % in autopsy series [6–14]. Patients with MC clinically present with subtle and heterogeneous signs and symptoms that depend on the anatomical site involved (cerebral hemispheres, cranial nerves, and/or spinal cord) [5, 15–17]. Diagnosis is based on cytological CSF examination and/or magnetic resonance imaging (MRI); these tests reportedly exhibit a sensitivity ranging from 45 to 80 % for CSF examination and from 20 to 91 % for MRI [5, 18]. Currently, therapeutic strategies include radiotherapy and intrathecal or systemic chemotherapy; however, the results remain poor because late diagnosis usually leads to non-eligibility of patients for treatment and to the delivery of palliative care only [19]. An earlier MC diagnosis could also improve the quality of life of BC patients who sometimes rapidly progress and need of institutional/hospital care. In fact, survival ranges from 4 to 6 weeks from diagnosis in untreated patients to 6 months when patients are immediately subjected to aggressive treatment [20, 21]. Longer survival is reported in only a few cases (13 % at 1 year and 6 % at 2 years) [16].

Few data are available that focus on the association of MC with peculiar clinico-morphological-molecular features in BC patients, and no studies has effectively pinpointed specific predictors of MC development. The most common parameters that have been identified to date are: young age, ≥4 metastatic lymph nodes, high histological tumor grade, HER2-positive *status* and triple negative immune-phenotype [10, 22–26]. Recently, a significant association with lobular histological type, estrogen receptor (ER) and progesterone receptor (PR)-negative *status* have been highlighted, as well as the presence of metastases at diagnosis [27].

In this context, the aim of this study is twofold: (1) to estimate the incidence of MC in a large cohort of consecutive and unselected BC patients who were treated in the same Breast Unit and (2) to define which clinico-pathological and molecular parameters, if any, are significantly associated with MC development in order to identify patients at high risk at an earlier stage.

## Methods

### Cohort definition and follow-up procedures

Our initial cohort included a consecutive series of BC cases comprising the entire population of 2017 patients who underwent surgery at the Città della Salute e della Scienza of Turin between 1998 and 2010.

For all patients, follow-up data were retrieved from the oncologists’ clinical records, and the last visit recorded in the cohort was registered on November 21st, 2013.

The study was submitted to and approved by the Ethic Institutional Review Board for “Biobanking and use of human tissues for experimental studies” of the Pathology Service of the Azienda Ospedaliera Città della Salute e della Scienza di Torino, Torino, Italy. The project provided a verbal and not written informed consent from the patients due to the retrospective approach of the study, which did not impact on their treatment. All the cases were anonymously recorded. The Institutional Review Board approved this consent procedure.

**Fig. 1:**
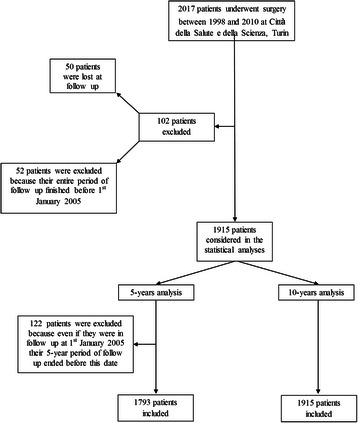
Patient series included in the study

To accurately estimate the MC incidence rate, the starting date for the analyses was set at January 1st, 2005, when MC started to be systematically recorded in the Department of Neuro-Oncology of our Hospital in BC population. Therefore, even though the follow up started at the time of surgery, just in the subgroup of patients who underwent surgery before the first of January 2005, this date was considered as the initial period of observation. The follow up ended at the last visit or at MC diagnosis. Consequently, from the initial cohort of 2017 patients, we excluded 50 patients without any follow up data (lost) and 52 patients whose follow up ended before the first of January 2005 (because MC was never investigated in this subgroup of patients). Therefore, 1915 patients were considered in further analyses (Fig. 1).

To enhance the study validity, we restricted the main analysis to the first 5 years of follow up after surgery, because most of the patients regularly participated in the scheduled visits during this initial period, with very limited loss during follow up. For this analysis, which was limited to the first 5 years of follow up, we excluded 122 additional patients who underwent surgery more than 5 years before the 1st of January, 2005, even though they were still in follow up at this time. Therefore, the final analysis, in which MC incidence rate was estimated during the first 5 years of follow up, included 1793 patients (Fig. 1).

As an additional study group, we considered the entire population of 1915 patients in an extended 10-year follow up (Fig. 1); in this analysis, MC incidence estimates are based on a larger number of events but are potentially biased due to lower compliance at the scheduled follow-up visits from 5 to 10 years after surgery. Further extension of the follow-up up to 15 years would introduce even greater bias, as all patients with no complications are referred to their general practitioner 10 years after BC diagnosis. Only patients with complications or disease recurrence (including MC) are still followed by the Breast Unit at the hospital.

### Clinical and pathological features

For each case, clinical, morphological, immunohistochemical and molecular data were collected. MC was suspected in patients upon initial neurological symptoms that are suggestive of meningeal involvement (i.e., headache, cranial nerve palsy, back pain, radicular pain, leg weakness, etc.). Clinical diagnoses were confirmed based on positive abnormal brain or spinal cord MRI and/or positive CSF cytology.

### Statistical analysis

All statistical analyses were conducted using the software STATA 11. We first estimated the incidence rate of MC, including 95 % confidence intervals (CI), over the first 5 years or the first 10 years of follow up after surgery for BC. The cumulative risks of MC over 5-year and 10-year after diagnosis of BC were estimated using Kaplan-Meier analysis.

In addition, despite the limited number of MC cases, the clinical, morphological and molecular characteristics of the case series were studied to determine whether any feature of the primary tumor was predictive of higher MC risk. Several a priori selected variables, including age, type of surgery, grading, lymph node involvement, tumor size, vascular invasion, ER and PR *status*, Ki-67 immunostaining and HER2 expression, were considered. Variables were categorized according to the literature and international guidelines [28–32]. Due to the very limited number of events, only univariate analyses were conducted.

## Results

The 1915 BC patients were women aged 22–93 (mean age 61.2 years) and included 338 patients (17.6 %) younger than 50 years. Clinical and morphological data regarding these women are reported in Table 1, column A.

**Table 1:** Clinico-pathological and molecular features of 1915 patients who were included in our analyses and underwent surgery between 1998 and 2010 (column A), highlighting the features of 6 patients with MC occurring during the first 10 years of follow up and considered in the analyses (column B)

Collected parameters	Column A	Column B
	Patients *n* = 1915 (%)	Patients with MC *n* = 6 (%)
Age at diagnosis of BC		
- <50 years	338 (17.6 %)	6 (100 %)
- ≥50 years	1577 (82.4 %)	0
Treatment		
- T (umorectomy)	1400 (73.1 %)	2 (33.3 %)
- M (astectomy)	515 (26.9 %)	4 (66.7 %)
Histological type		
- IDC	1154 (60.3 %)	5 (83.3 %)
- ILI	348 (18.2 %)	0
- Mixed IDC-ILI	146 (7.6 %)	1 (16.7 %)
- Others	267 (13.9 %)	0
Histological grade		
- G1	564 (29.5 %)	0
- G2	908 (47.4 %)	2 (33.3 %)
- G3	435 (22.7 %)	4 (66.7 %)
- Not available	8 (0.4 %)	0
Tumor size		
- ≤ 15 mm	1019 (53.2 %)	0
- > 15 mm	890 (46.5 %)	6 (100 %)
- Not available	6 (0.3 %)	0
Vascular invasion		
- No	1271 (66.4 %)	1 (16.7 %)
- Yes	626 (32.7 %)	5 (83.3 %)
- Not available	18 (0.9 %)	0
pT stage		
- pT1	1342 (70.1 %)	1 (16.7 %)
- pT2	493 (25.7 %)	5 (83.3 %)
- pT3-4	76 (4 %)	0
- Not available	4 (0.2 %)	0
pN stage		
- Negative (including ITC/micrometastasis)/pN0	1335 (69.7 %)	1 (16.7 %)
- from 1 to 3 nodes positive/pN1	338 (17.7 %)	2 (33.3 %)
- >3 nodes positive/pN2-pN3	167 (8.7 %)	3 (50 %)
- Not available	75 (3.9 %)	0
cM stage at diagnosis		
cM0	1889 (98.6 %)	4 (66.7 %)
cM1	26 (1.4 %)	2 (33.3 %)
Bone metastases		
- no	1818 (94.9 %)	3 (50 %)
- yes	97 (5.1 %)	3 (50 %)
Brain metastases		
- no	1898 (99.1 %)	1 (16.7 %)
- yes	17 (0.9 %)	5 (83.3 %)
Metastases in other site, NAS		
- no	1686 (88 %)	0
- yes	229 (12 %)	6 (100 %)
ER *status*		
Negative	190 (9.9 %)	3 (50 %)
Positive	1693 (88.4 %)	3 (50 %)
Not available	32 (1.7 %)	0
PR *status*		
- Negative	256 (13.4 %)	2 (33.3 %)
- Positive	1365 (71.3 %)	3 (50 %)
- Not available	294 (15.4 %)	1 (16.7 %)
Ki67		
- <20 %	1103 (57.6 %)	2 (33.3 %)
- ≥20 %	741 (38.7 %)	4 (66.7 %)
- Not available	71 (3.7 %)	0
HER2		
- negative	1662 (86.8 %)	3 (50 %)
- positive	151 (7.9 %)	3 (50 %)
- Not available	101 (5.3 %)	0
Neoadjuvant chemotherapy		
- No	1836 (95.9 %)	3 (50 %)
- Yes	79 (4.1 %)	3 (50 %)
Adjuvant treatment		
- Chemotherapy	722 (37.7 %)	4 (66.7 %)
- Radiotherapy	1352 (70.6 %)	1 (16.7 %)
- Hormone therapy	1520 (79.4 %)	2 (33.3 %)

*IDC* Infiltrating ductal carcinoma, *ILC* Infiltrating lobular carcinoma, *G* histological tumor grade [28], *ITC* isolated tumor cells

Overall, we identified nine cases of MC out of 1915 BC patients. Three out of nine patients developed leptomeningeal involvement more than 10 years after their primary BC surgery and were therefore excluded from the analyses. The clinico-pathological features of the 6 patients with MC included in this study are summarized in Table 1, column B.

### Incidence and risk of MC

The median follow up period for the 1793 patients included in the analyses that were limited to the first 5 years of follow up was 4.78 years, and 47 % of the patients were followed up for the full 5 years. When analyses were prolonged until 10 years of follow up (1915 patients), the median follow up period was slightly increased to 5.03 years, and only 7 % of the patients were followed up for the full 10-year period.

In the first 5 years of follow up, three patients developed MC, corresponding to a rate of 5.44 per 10,000 patients per year (95 % CI: 1.75–16.9) and an overall 5-year risk of 0.3 % (95 % CI: 0.1–0.8) (Table 2, column A). Extending the period of observation to 10 years of follow up revealed a rate of 7.55 per 10,000 patients per year (0.6 % 10-year risk, 95 % CI: 0.3–1.4), based on six MC patients.

**Table 2:** Rate of breast cancer-related meningeal carcinomatosis, 5 years (column A) and 10 years (column B) after surgery

	Column A: 5-year follow up^a^				Column B: 10-year follow up^b^			
	MC events	Patients	Person-years	Rate (per 10.000)	MC events	Patients	Person-years	Rate (per 10.000)
Patients	3	1793	5518	5.44 (1.75–16.9)	6	1915	7947	7.55 (3.39–16.8)
Age at diagnosis								
- <50 years	3	324	999	30.0 (9.69–93.1)	6	338	1446	41.1 (18.6–92.4)
- ≥50 years	0	1469	4519	0	0	1577	6502	0
				(***p*** =**0.002**)				(***p*** < **0.001**)
Treatment								
-Tumorectomy	2	1325	4146	4.82 (1.21–19.3)	2	515	1972	20.3 (7.61–54.0)
-Mastectomy	1	468	1372	7.29 (1.03–51.7)	4	1400	5975	3.34 (0.84–13.4)
				(*p* =0.73)				(***p*** = **0.018**)
Histological grade								
- G1	0	528	1515	0	0	564	2323	0
- G2	0	854	2770	0	2	908	3799	5.26 (1.32–21.0)
- G3	3	403	1211	24.8 (7.99–76.8)	4	435	1794	22.3 (8.4–59.4)
				(***p*** < **0.001**)				(***p*** = **0.007**)
Tumor size								
- ≤ 15 mm	0	949	2927	0	0	1019	4280	0
- > 15 mm	3	838	2581	11.6 (3.75–36.0)	6	890	3649	16.4 (7.39–36.6)
				(***p*** = **0.07**)				(***p*** = **0.008**)
Vascular invasion								
- No	1	1204	3779	2.64 (0.37–18.8)	1	1271	5323	1.88 (0.26–13.3)
- Yes	2	574	1697	11.8 (2.95–47.1)	5	626	2558	19.5 (8.13–47.0)
				(*p* = 0.18)				(***p*** = **0.008**)
pT stage								
- pT1	1	1262	3945	2.53 (0.36–18.0)	1	1342	5683	1.76 (0.25–12.5)
- pT2	2	461	1394	14.3 (3.59–57.4)	5	493	1984	25.2 (10.5–60.5)
- pT3-4	0	66	172	0	0	76	269	0
				(*p* = 0.30)				(***p*** = **0.01**)
pN stage								
- Negative (including ITC/micrometastasis)/pN0	1	1273	3981	2.51 (0.35–17.8)	1	1335	5594	1.79 (0.25–12.7)
- from 1 to 3 positive/pN1	1	319	1006	9.94 (1.40–70.6)	2	338	1452	13.8 (3.44–55.1)
- >3 positives/pN2-pN3	1	149	391	25.6 (3.61–182)	3	167	604	49.7 (16.0–154)
				(*p* = 0.26)				(***p*** = **0.004**)
ER *status*								
- Negative	2	181	532	37.6 (9.40–150)	3	190	776	38.6 (12.5–119)
- Positive	1	1581	4869	2.05 (0.89–14.6)	3	1693	7015	4.28 (1.38–13.3)
				(***p*** = **0.001**)				(***p*** = **0.001**)
PR *status*								
- Negative	2	231	653	15.3 (2.16–109)	2	256	966	10.4 (1.46–73.5)
- Positive	1	1270	4090	4.89 (1.22–19.6)	3	1365	5496	7.28 (2.73–19.4)
				(*p* = 0.054)				(*p* = 0.19)
Ki67								
- <20 %	0	1041	3224	0	2	1103	4600	4.35 (1.09–17.4)
- ≥20 %	3	685	2037	14.7 (4.75–45.7)	4	741	2990	13.4 (5.02–35.6)
				(***p*** = **0.03**)				(*p* = 0.17)
HER2								
- Negative	1	1574	4875	2.05 (0.29–14.6)	3	1662	6867	4.37 (1.41–13.5)
- Positive	2	144	452	44.2 (11.01–177)	3	151	632	47.5 (15.3–147)
				(***p*** < **0.001**)				(***p*** < **0.001**)

*ITC* isolated tumor cells

^a^missing data: Grading: 8 missing; pN stage: 52 missing; tumor size: 6 missing; ER: 31 missing; PR: 292 missing; Ki67: 67 missing; Vascular invasion: 15 missing; pT stage: 4 missing; HER2: 75 missing

^b^missing data: Grading: 8 missing; pN stage: 75 missing; tumor size: 6 missing; ER: 32 missing; PR: 294 missing; Ki67: 71 missing; Vascular invasion: 18 missing; pT stage: 4 missing; HER2: 101 missing

### Clinico-pathological features and MC development

Among the 6 patients who developed MC, the median age at time of BC diagnosis was 39.5 years (range, 32 to 49 years) and all patients showed a tumor size larger than 15 mm. The most common histological subtype was infiltrative ductal carcinoma (5/6 cases) with a high (66.7 %) or intermediate (33.3 %) histological tumor grade. Three of the six patients (50 %) had more than three axillary lymph node metastases. ER and PR immunostain was positive in 50 % of cases; in 66.7 % of BC cases, Ki67 was higher than 20 %. HER2 *status* was positive in 3/6 cases (50 %). Vascular invasion was present in five cases (83.3 %).

Univariate analysis showed that younger age, tumor size more than 15 mm, high histological tumor grade (G3), ER-negative *status*, HER2-positive *status* and high proliferative index were associated with MC development, considering the first 5 years of follow up (Table 2, column A). These associations were replicated in the analyses based on 10 years of follow-up extended analysis; in the latter analyses, the higher pT, more than three metastatic lymph nodes and presence of vascular invasion were also associated with an increased risk of MC (Table 2, column B).

Finally, it appears that the rate of MC was higher in the subgroup of patients with bone and/or brain metastases (Table 3).

**Table 3:** Rate of breast cancer related to meningeal carcinomatosis in patients with bone and/or brain metastasis occurrence: 5 years (column A) and 10 years (column B) after surgery

	Column A: 5-year follow up				Column B: 10-year follow up			
	MC events	Patients	Person-years	Rate (per 100)	MC events	Patients	Person-years	Rate (per 100)
Occurrence of bone metastasis^a^								
No	2	1780	5434	0.037 (0.0092–0.147)	3	1898	7770	0.039 (0.012–0.12)
Yes	1	52	84	1.19 (0.168–0.85)	3	87	177	1.69 (0.55–5.25)
				(***p*** < **0.001**)				(***p*** < **0.001**)
Occurrence of brain metastasis								
No	2	1792	5510	0.036 (0.0091–0.15)	3	1912	7929	0.038 (0.012–0.12)
yes	1	9	7	14.36 (2.02–101.93)	3	15	18	16.58 (5.35–51.41)
				(***p*** < **0.001**)				(***p*** < **0.001**)

^a^Patients sum up to more than 1793 (5-year follow-up) or 1915 (10-year follow-up) because bone and brain metastases are time-dependent variables; thus, a single patient who developed metastasis during the follow up contributed to both levels (yes and no) of the variable

## Discussion

MC diagnosis has increased in recent years due to advances in imaging technology and the development of new chemotherapy and immunotherapy approaches that have improved the survival of patients with BC. Despite the observed increase in clinical practice [27, 33], our results suggest that the incidence of MC remains low, when considering an unselected population undergoing surgery for BC. To date, few studies have analyzed the occurrence of MC in patients with BC, and the results vary depending on the study approach used. Briefly, in advanced stages and autopsy series, MC incidence was observed with rates ranging from 2.6 to 16 % [9, 11–14]. In a retrospective study based on a selected subgroup of BC patients with metastatic disease, Yap et al. reported a 5 % incidence [6]. More recently, in a series of BC with HER2 overexpression, MC incidence was observed in up to 6.6 % of cases [8]. However, these relevant percentages need to be critically interpreted because they have been observed in selected high-risk categories of patients with BC. The question then arises, what is the incidence of MC in an unselected population with BC? To address this issue, we decided to apply a rigorous definition of our BC cohort to avoid selection bias. In fact, we included all patients with BC who were consecutively treated in our Breast Unit, not only those with metastatic/advanced disease or with high-risk parameters. In addition, we first restricted the main analyses to patients who were expected to attend with high compliance during the periodic follow up; that is, the large subgroup of patients who regularly attended scheduled visits (approximately every 6 months) in the first 5 years after BC diagnosis and surgery, regardless to disease stage.

In this well-defined cohort, 3 patients developed MC, with a rate of incidence of 5.44 per 10,000 per year and a 5-year risk of 0.3 %. Because some studies have reported that the incidence of MC is most common in patients with BC after 10 years from diagnosis [15, 34], we also conducted an additional 10-year follow up analysis. During this period, we registered 3 additional cases of MC, which determined a non-substantial increase in the rate of MC incidence. Overall, in our consecutive and unselected population of patients with BC who were treated and followed in the same Breast Unit, the 5- and 10-year risk rates of MC development were lower than 1 %. Similarly, brain metastases occurred in 0.9 % of the cohort (and 83 % of MC exhibited an associated brain lesion). These results appear similar to those reported two decades ago by Jayson et al., who described a 0.86 % incidence rate of MC in an unselected series of patients with BC [7]. In the same study, focusing on a subgroup of patients treated for recurrent disease, an increased incidence of up to approximately 2 % was observed [7].

To our knowledge, this was the only study to examine the unbiased incidence rate of MC in BC, and the consistency of the results with our data obtained 20 years later leads to two hypotheses: either the “real” incidence of MC in an unselected BC population has undergone an apparent, but not true increase, or the “real” incidence remains underestimated.

Considering that patients with BC and neurological symptoms may be referred to the emergency room or to a neurological setting (where MC is usually managed), we can hypothesize that the source of the data (Breast Unit medical charts) might represent a limitation.

As a second step, we attempted to solve the important and partially unmet need to identify patients with BC who are at high risk for MC development; to this end, we searched for clinico-pathological and molecular features that might be useful as possible predictors of increased MC risk. We observed that established predictors of worse prognosis (young age, tumor size exceeding 15 mm, high histological grade, more than three metastatic lymph nodes, ER-negative and HER2-positive *status* and high proliferative index) were associated with an increased risk of MC, in agreement with other studies [25, 27, 35–39].

## Conclusions

MC remains a rare condition with a usual occurrence in the natural history of BC. Late diagnosis of MC leads patients to non-eligibility for standard therapeutic strategies, including radiotherapy and intrathecal and/or systemic chemotherapy and to the delivery of palliative care. Consequently, the identification of a clinico-pathological profile of patients with BC who are at increased MC risk would be useful so that they might be directed to a strict follow up aimed at anticipating MC diagnosis prior to symptom presentation, as also suggested by a recent review of clinical trials [40]. However, the low incidence of MC and the lack of specific risk factors make it difficult to generate a dedicated diagnostic screening work up that is designed to precociously detect MC in patients with BC.

## Abbreviations

BC: Breast Cancer
CSF: Cerebrospinal fluid
ER: Estrogen Receptor
MC: Meningeal Carcinomatosis
MRI: Magnetic Resonance Imaging
PR: Progesterone Receptor
